# Non-Retinotopic Motor-Visual Recalibration to Temporal Lag

**DOI:** 10.3389/fpsyg.2012.00487

**Published:** 2012-11-19

**Authors:** Masaki Tsujita, Makoto Ichikawa

**Affiliations:** ^1^Graduate School of Humanities and Social Sciences, Chiba UniversityChiba, Japan; ^2^Faculty of Letters, Chiba UniversityChiba, Japan

**Keywords:** point of subjective simultaneity, temporal lag adaptation, temporal order judgment, retinotopic specificity, method of constant stimuli

## Abstract

Temporal order judgment (TOJ) between the voluntary motor action and its perceptual feedback is important in distinguishing between a sensory feedback which is caused by observer’s own action and other stimulus, which are irrelevant to that action. Prolonged exposure to fixed temporal lag between motor action and visual feedback recalibrates motor-visual temporal relationship, and consequently shifts the point of subjective simultaneity (PSS). Previous studies on the audio-visual temporal recalibration without voluntary action revealed that the low level processing is involved. However, it is not clear how the low and high level processings affect the recalibration to constant temporal lag between voluntary action and visual feedback. This study examined retinotopic specificity of the motor-visual temporal recalibration. During the adaptation phase, observers repeatedly pressed a key, and visual stimulus was presented in left or right visual field with a fixed temporal lag (0 or 200 ms). In the test phase, observers performed a TOJ for observer’s voluntary keypress and test stimulus, which was presented in the same as or opposite to the visual field in which the stimulus was presented in the adaptation phase. We found that the PSS was shifted toward the exposed lag in both visual fields. These results suggest that the low visual processing, which is retinotopically specific, has minor contribution to the motor-visual temporal lag adaptation, and that the adaptation to shift the PSS mainly depends upon the high level processing such as attention to specific properties of the stimulus.

## Introduction

Temporal order judgment (TOJ) between motor action and perception is important in distinguishing between a sensory feedback resulting from observer’s own action and a lot of external events, which are irrelevant to the action. For instance, on the one hand, if we perceive a clapping sound simultaneously with the motor information of our own clapping hands, we will feel that the sound is caused by our own action. On the other hand, if we perceive the clapping sounds before or after our own clapping motion, we will feel the sound is caused by the other person’s clapping action. Because temporal aspect of our perception depends upon several factors, such as stimulus intensity (Lit, 1949; Anstis, 2001), eccentricity (Mitrani et al., 1986; Carrasco et al., 2003), and attention to stimulus (Posner, 1980; Hikosaka et al., 1993), there could be temporal discrepancy between the motor action and its perceptual feedback. Therefore, our perceptual system should compensate such temporal discrepancy for accurate TOJ between motor action and perceptual signals, and for accurate detection the perceptual event, which is caused by our own motor action.

As one of the way to compensate the discrepancy between motor signal and perceptual feedback, our perceptual system can recalibrate the motor-sensory temporal relationship. Stetson et al. (2006) reported the temporal recalibration between motor action and visual feedback. In their experiment, their observers were exposed to the consistent 100 ms injected lag between a keypress and a subsequent visual flash. After a few minutes of the exposure, they performed a TOJ task between keypress and flash. Consequently, the point of subjective simultaneity (PSS) was shifted toward the direction to compensate the lag.

Several studies have examined whether the temporal recalibration between the observer’s own action and its perceptual feedback is modality specific. For instance, Heron et al. (2009) demonstrated that the temporal recalibration after the exposure to the temporal lag between the mousepress and its perceptual feedback was not restricted to the vision. That is, they found the temporal recalibration not only between mousepress and visual flash but also between mousepress and auditory or tactile stimulus after the exposure to the delay of sensory feedback ranging from 50 to 800 ms. Moreover, Sugano et al. (2010) revealed that the temporal recalibration between finger taps and the stimulus to one of the perceptual modalities (e.g., vision) can be generalized to the temporal relationship between motor action and stimulus to the other perceptual modality (e.g., audition). Although these studies have demonstrated that the temporal recalibration between observer’s own motor action and its perceptual feedback is robust and consistent phenomenon, these studies did not reveal the processing which underlies the motor-sensory temporal recalibration.

Several studies have shown that some perceptual adaptations, which are related to temporal aspects of visual stimulus, are specific to the retinal position where the adaptation stimulus was presented. For instance, Bruno et al. (2010) revealed that the reduction of apparent duration, which was obtained during exposure to oscillating motion or flicker for 20 or 45 s, is restricted to the retinal position where the oscillating motion or flicker stimulus was presented as adaptation stimulus. Melcher (2005) reported that the tilt aftereffect, form aftereffect, and face aftereffect were not restricted to the retinal position where the adaptation stimuli were presented, while the contrast adaptation was retinotopically specific. These results indicate that the retinotopic specificity in adaptation depends upon the attributes to which the perceptual system adapted, and therefore, upon the processing which underlies the processing of those attributes. The retinotopic specificity in perceptual adaptation can be considered as an evidence of involvement of the low-level processing because the neural response of early visual area including primary and secondary cortex well corresponds to the stimulus to the specific retinal positions (e.g., Duhamel et al., 1997).

The retinotopic specificity has been found in the perceptual learning concerning with the perceptual tasks which depend on the low-level processing, such as orientation discrimination (Schoups et al., 1995), texture discrimination (Karni and Sagi, 1991), depth detection (Ramachandran, 1976; O’Toole and Kersten, 1992), and so on. Moreover, the temporal recalibration between audio and visual stimuli, which is caused by prolonged exposure to a consistent temporal lag between audio and visual stimuli (Fujisaki et al., 2004; Vroomen et al., 2004), is specific to the retinal position where the visual stimulus was presented during the adaptation (Heron et al., 2012). That is, during the adaptation period, two visual stimuli were presented at different retinal positions. The temporal lag between the visual and auditory stimuli varied with the retinal positions. After the adaptation period, they found that shift of PSS at each retinal position varied with the temporal lag which was inserted at that retinal position during the adaptation period. From results of these previous studies, one may expect that such a retinotopically specific, and therefore low-level processing is involved in the temporal recalibration between the active keypress and visual stimulus.

In this study, we investigated the basis of the motor-visual recalibration by examining whether the temporal recalibration in terms of the motor-visual lag was specific to the retinal position. If the low-level processing is responsible to the motor-visual temporal recalibration, the effects of recalibration would be observed only in the retinal position where the visual stimulus was presented during the adaptation. If the high level processing is responsible, the effects of recalibration would be observed regardless of the retinal position.

## Materials and Methods

### Observers

Eight observers (six right-handed and two left-handed) including one author participated to the experiment. All had normal or corrected-to-normal vision.

### Stimuli and apparatus

Observers sat at a desk in a dimly lit room and looked at a 19-inch CRT display (EIZO FlexScan T766) with a refresh rate of 60 Hz at 57 cm viewing distance. As a fixation point, a white cross (1 × 1 arc deg, 107.5 cd/m^2^) was presented at the center of the display.

As an adaptation stimulus, a white square (1 × 1 arc deg, 107.5 cd/m^2^) flashed for one flame on a black background (0.1 cd/m^2^) about 4.5 arc deg left or right from the fixation point. As a test stimulus, the same white square was flashed for one flame at the same position as, or the opposite position to the adaptation stimulus.

**Figure 1:**
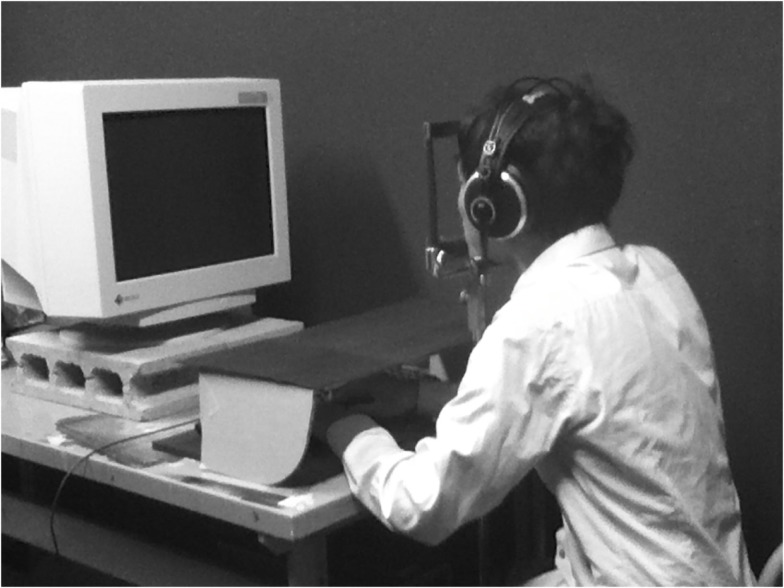
**Apparatus**. Observers couldn’t hear keypresses because of white noise via headphone and couldn’t, see their hands because of a cover.

Observers used a keyboard (Dell SK-8175 keyboard) for adaptation task and for TOJ task. The keyboard was located on the desk. Observers’ hands were covered so that they could not see their own hands during the experiment. Observers wore a headphone (AKG K271 MKII) which presented white noise in order not to hear sounds pressing the keyboard (Figure 1).

### Procedure

**Figure 2:**
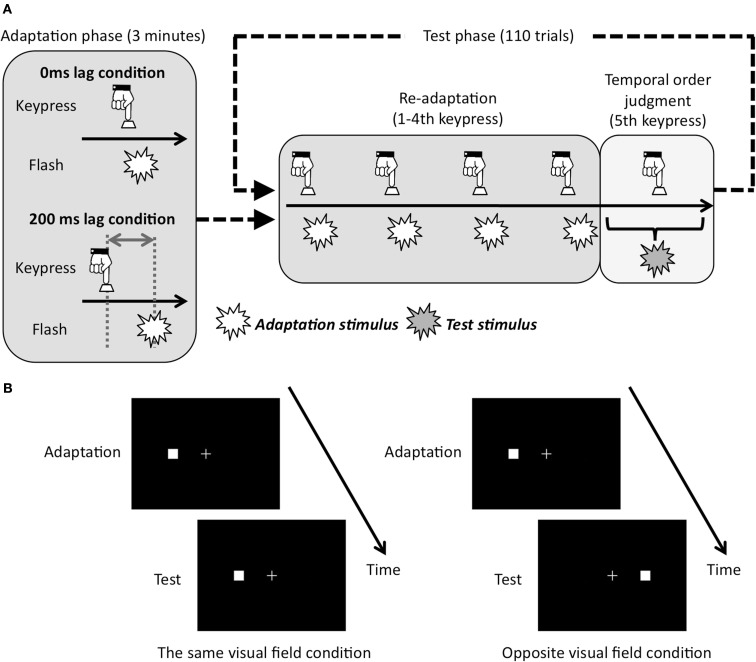
**Schematic showing of experimental conditions and procedure**. **(A)** Adapted lag conditions and experimental run. Conditions of re-adaptation were corresponded with conditions of adaptation phase. **(B)** Test visual field conditions. In case the adapted visual field was left, the test visual field was left in the same visual field condition, and was right in opposite visual field condition.

Experimental sessions were composed of adaptation phase and test phase (Figure 2A). The adaptation phase preceded the test phase. At the beginning of the adaptation phase, in order to show the adequate pace for the keypress to observers, thickness of the fixation cross changed three times from 0.2 to 0.4 arc deg for one flame with 1.54 Hz. After that, observers tried to repeatedly press the key by the use of their right hand with the same pace as the initial change of the fixation cross for 3 min (averaged 284 keypresses). There were two temporal lag conditions for the visual feedback stimulus. That is, observers were exposed to the adaptation stimuli that were presented in each keypress with 0 or 200 ms lag at right or left visual field. We used these temporal lag conditions because a previous study (Heron et al., 2009), which used the temporal lag ranging from 50 to 800 ms, reported that the magnitude of PSS shift caused by motor-visual temporal recalibration was largest at 200 ms temporal lag. Adapted visual field was counterbalanced across blocks.

Each trial in the test phase began with a re-adaptation period. In the re-adaptation period, the thickness of the fixation cross changed three times with 1.54 Hz to show the adequate pace of the keypress. By following the change of thickness in fixation cross, the observers tried to press the key five times with a constant pace. For the first four keypresses, the visual flashes as adaptation stimuli were presented with a consistent temporal lag from the keypress in either the same as or opposite to the adapted visual field. For the last (fifth) key press, observers conducted TOJ task. That is, the fifth visual stimulus was presented with 11 temporal lag conditions (0, ±30, ±60, ±90, ±120, and ±150 ms) from the plausible timing of the fifth keypress, which were derived from the averaged interval for the first four keypresses. After the fifth visual flash was presented, observers judged whether the fifth visual flash was before or after the fifth keypress.

Two within-subject factors were used: adapted lag between a keypress and a visual flash during adaptation phase (0 or 200 ms lag: Figure 2A), test visual field during test phase (the same or opposite visual field: Figure 2B). The experimental sessions for each condition were composed of four blocks. Each block included 3 min of adaptation phase, and following 110 trials for the TOJ task. The whole test consisted of 1760 trials with 440 repetitions for each of the four conditions. Because four blocks with different conditions were conducted per day, the experiment took 4 days for each individual. These factors were fixed in each block and counterbalanced across observers. In each block, the order of the temporal lag condition was random in the test phase. To be familiar with the procedure, before the experimental session, each observer had a practice session, which was composed of exposure to the adaptation stimulus for 20 s with 0 ms lag and 10 trials of TOJ task.

This study was approved by ethical committee of the department.

## Results

**Figure 3:**
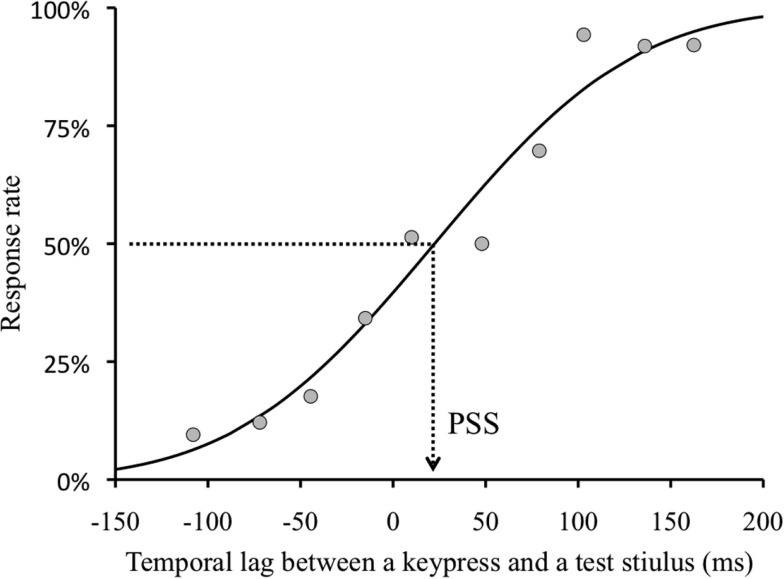
**Sample psychometric function**. Example of an observer H. I. for 0 ms lag and opposite visual field condition. The dots indicate the proportion of “A visual flash appeared after a keypress.” response in each divided group. Negative value of temporal lag indicates that a test stimulus was presented before a keypress.

We obtained a TOJ response and a temporal lag between the real fifth keypress and a test stimulus (negative value indicates that the test visual stimulus was presented before the keypress) in each trial. As TOJ response, we used the frequency in the trial in which the visual flash was perceived after the keypress. We binned the TOJ response by the temporal lag between the fifth keypress and visual flash (30 ms bin). We found no significant effect of adapted visual field (right or left visual field). Therefore, the results of this condition were combined in the following analyses. In order to obtain the temporal lag between the keypress and visual flash with which the observers perceived the keypress as simultaneous with the visual flash, we conducted a Probit analysis (see Finney, 1971) for individual observers’ data of each condition. We obtained 50% threshold as PSS (Figure 3) for each condition.

**Figure 4:**
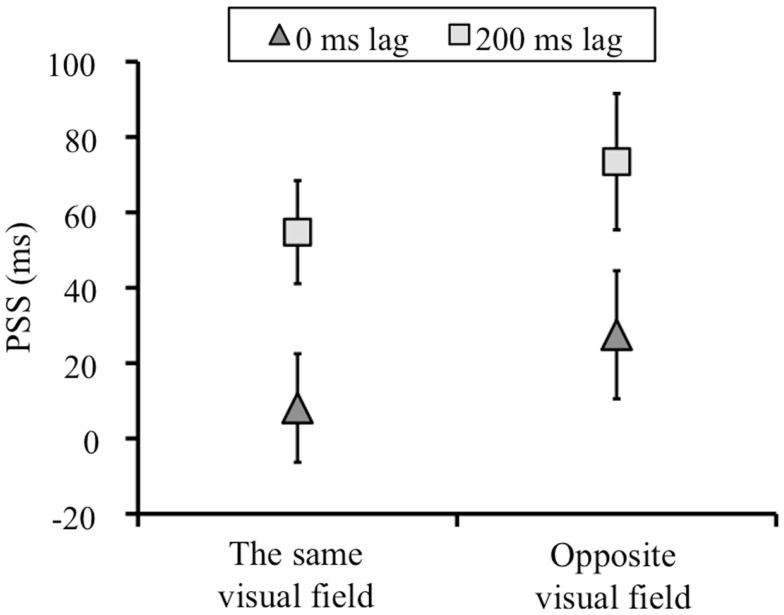
**The means of the PSSs (ms) in each condition**. Negative values in PSS indicates a test stimulus before a keypress. Error bars show standard error of mean.

Figure 4 showed the means of the PSSs for each condition. The PSSs shift between 0 and 200 ms lag condition in the direction of the lag means that the temporal order perception between keypress and visual flash was recalibrated by the temporal lag adaptation. We conducted a two-way repeated measures analysis of variance (ANOVA) with the adapted lag (0 and 200 ms) and visual field (left and right) as factors for on the PSSs and slopes. For the PSSs, we found significant main effect of adapted lag [*F*(1, 7) = 17.346, *p* = 0.004]. The main effect of test visual field [*F*(1, 7) = 5.082, *p* > 0.05] and the interaction between the two factors [*F*(1, 7) = 1.060, *p* > 0.05] was not significant. That is, the PSSs shifted in both the same and opposite visual field condition, and there was no difference in the magnitude of the PSSs shift between the same and opposite visual field condition (Figure 4).

For the slopes of psychometric function, which corresponds the precision in temporal order perception between keypress and visual flash, we found no significant main effect or interaction. These results indicate that the difference of adapted lag and test visual field in the motor-visual temporal lag adaptation didn’t affect the temporal order sensitivity.

## Discussion

We found that, after exposure to temporal lag between the keypress and visual stimulus for a few minutes, observers obtained the motor-visual temporal lag adaptation not only in the same visual field, but also in opposite visual field. These results indicate that the motor-visual temporal recalibration is not restricted to the retinal position where the visual feedback stimulus is presented with a temporal lag. In Introduction, we have seen that many types of adaptation and visual learning are restricted to the retinal position where the visual stimulus was presented during the adaptation period, and that low-level processing is responsible for those adaptation and perceptual learning. However, the present results imply that, not the retinotopically specific low-level processing, but higher level processing would be responsible for the motor-visual temporal recalibration.

It is open to debate whether the processing which underlies the motor-sensory temporal recalibration is the same as or different from the processing which underlies the multisensory (e.g., audio-visual) temporal recalibration (Fujisaki et al., 2004; Vroomen et al., 2004; Heron et al., 2012). When clapping hands, we would acquire both copies of motor signal (the so-called efference copy; e.g., von Helmholtz, 1867/1962; Bridgeman, 1995) and various sensory signals as feedbacks to the hands movement (such as visual, auditory, kinesthetic, and tactile signals from the hand). Whereas, when exposing an event without our own action, we would acquire only sensory signals from the event. This difference in acquired signals between the motor-sensory and the multisensory temporal recalibration would cause different properties for those recalibrations.

Previous studies have not revealed the obvious difference between the motor-sensory and multisensory temporal recalibration, but rather shown similarity between them. For instance, Sugano et al. (2010) demonstrated that the temporal recalibration between motor and vision (or audition) could be transferred to the temporal relationship between motor and audition (or vision). Similar transfer of temporal recalibration across the perceptual modality was also found in the multisensory temporal recalibration (Di Luca et al., 2009). One may assume that the process which underlies the motor-sensory temporal recalibration is similar to the process which underlies the multisensory temporal recalibration, and that these two processes share some common stages.

However, the present results suggest that the motor-sensory temporal recalibration is based upon the processing which is different from the processing for the multisensory temporal recalibration. That is, we revealed the strong evidence for the notion that the mechanism which underlies the motor-visual temporal recalibration is different from the mechanism which underlies the audio-visual temporal recalibration. On the one hand, as we referred in Introduction, the audio-visual temporal recalibration is restricted to the retinal position where the visual stimulus was presented during the adaptation (Heron et al., 2012). On the other hand, the present results showed that the motor-visual temporal recalibration was not restricted to the adapted retinal position. This difference indicates that the processing for the motor-sensory recalibration is relatively higher than the processing for the multisensory recalibration which is retinotopically specific.

For the opposite visual field condition, the eccentricity of the adaptation stimulus in the visual field was about 4.5 arc deg, and the retinal position of the test stimulus was about 9 arc deg distant from that of the adapted retinal position. Physiological studies have revealed that size of receptive field in visual cortex increased systematically in proportion to the order of visual area. That is, the size of receptive field of neurons in human V1 and V2, which are tuned to specific spatiotemporal features, and which are modulated by visual attention, was less than 2 arc deg at 4.5 arc deg of eccentricity (e.g., Dumoulin and Wandell, 2007; Amano et al., 2009). Therefore, one may infer that higher area than at least V1 and V2 is involved in the motor-visual temporal recalibration. Moreover, we should note that the adaptation was not restricted to the specific hemi-visual field to which the adaptive stimulus was presented. This result implies that the motor-visual temporal recalibration is related to the higher processing which may involve both of right and left brain hemispheres.

An important difference between the motor-sensory temporal recalibration and multisensory temporal recalibration is concerning with the activity of observers during the experimental sessions. That is, on the one hand, for the motor-visual temporal lag adaptation, observers would actively obtain the visual feedbacks by more precisely predicting during the adaptation period in which they voluntarily moved their own hand. On the other hand, for the audio-visual temporal lag adaptation (Heron et al., 2012), observers would be passively exposed to the audio-visual temporal lag, which is independent of observer’s voluntary behavior. Previous study revealed that, if observer voluntarily determines the presentation of stimulus, and therefore if the observer can predict the timing of the stimulus, visual attention may affect the temporal aspect of visual processing, and reduce the attentional blink deficit (Kihara and Kawahara, 2012), and illusory flash-lag effect (Lopez-Moliner and Linares, 2006). Recently, we found that removing attention from the visual stimulus, and allocating it to the auditory stimulus during the adaptation period, would reduce the motor-sensory temporal recalibration (Tsujita and Ichikawa, 2012). This finding supports the idea that voluntary attention to the visual stimulus is involved in the motor-sensory temporal recalibration. We are proposing that observer’s voluntary attention, which is accompanied in observer’s active movement during the adaptation plays an important role in the motor-visual temporal recalibration, and that this voluntary attention differentiates the motor-visual temporal recalibration from the multisensory temporal recalibration, which is mainly based on involuntary low-level processing.

We consider that the voluntary attention is essential for adopting temporal relationship between an efference copy and a specific sensory feedback in the motor-sensory temporal recalibration. As mentioned above, during an action, observers acquire its efference copy, and various sensory feedbacks. That is, in the present experiment, observers would acquire not only the visual feedbacks but also kinesthetic and tactile feedback from their hands and fingers. Although the efference copy is accompanied with various feedbacks in different modalities, observers would recalibrate temporal relationship between the efference copy and a sensory feedback to which the observers voluntarily attended. These considerations are compatible with the present results, and results of our recent study in which we found that removing attention from the visual stimulus during the adaptation period reduced the motor-sensory temporal recalibration (Tsujita and Ichikawa, 2012).

In the current study, the PSSs shift was approximately 23% of the temporal lag between the keypress and visual stimulus during the adaptation period. The extent of shift was relatively smaller than the shifts found in the previous studies (e.g., 44% in Stetson et al., 2006; 31% in Sugano et al., 2010). This relatively small shift would be caused by the temporal frequency of keypress. In this study, observers were instructed to repeatedly press the key with the same pace as the initial change of the fixation cross with 1.54 Hz, which was higher than the frequency used in Sugano et al. (2010; 1.33 Hz). Another plausible cause for the relative small shift is the spatial separation from the fixation point to the retinal position where the visual stimulus was presented during the adaptation period. In this study, the eccentricity of the visual stimulus was 4.5 arc deg although, in the other studies, the visual stimulus was presented in fovea. How temporal frequency of keypress and eccentricity affect the PSS shift in the motor-visual temporal recalibration should be examined in future study.

In summary, we examined retinotopic specificity of the motor-visual temporal recalibration. The motor-visual temporal recalibration should play an important role in the detection of visual stimulus which is caused by observer’s own motor action. The present results showed that the temporal recalibration occurred independently of adapted retinal position. These results suggest that higher level of processing that is related to attention to the stimulus would be involved in the motor-visual temporal recalibration. Future studies concerning with the perception of causality in motor action and consequential perceptual feedback have to examine how the low-level and high level of processings are involved in the motor-visual temporal recalibration.

## Conflict of Interest Statement

The authors declare that the research was conducted in the absence of any commercial or financial relationships that could be construed as a potential conflict of interest.
